# Automatic Segmentation of Type A Aortic Dissection on Computed Tomography Images Using Deep Learning Approach

**DOI:** 10.3390/diagnostics14131332

**Published:** 2024-06-23

**Authors:** Xiaoya Guo, Tianshu Liu, Yi Yang, Jianxin Dai, Liang Wang, Dalin Tang, Haoliang Sun

**Affiliations:** 1 School of Science, Nanjing University of Posts and Telecommunications, Nanjing 210023, China; tianshu271@163.com (T.L.); yang61yi@163.com (Y.Y.); daijx_js@126.com (J.D.); 2 School of Biological Science and Medical Engineering, Southeast University, Nanjing 210096, China; liangwang@seu.edu.cn (L.W.); dtang@wpi.edu (D.T.); 3 Mathematical Sciences Department, Worcester Polytechnic Institute, Worcester, MA 01609, USA; 4 Department of Cardiovascular Surgery, First Affiliated Hospital of Nanjing Medical University, Nanjing 210029, China

**Keywords:** type A aortic dissection, deep learning, computed tomography, image segmentation, nnU-Net

## Abstract

Purpose: Type A aortic dissection (TAAD) is a life-threatening aortic disease. The tear involves the ascending aorta and progresses into the separation of the layers of the aortic wall and the occurrence of a false lumen. Accurate segmentation of TAAD could provide assistance for disease assessment and guidance for clinical treatment. Methods: This study applied nnU-Net, a state-of-the-art biomedical segmentation network architecture, to segment contrast-enhanced CT images and quantify the morphological features for TAAD. CT datasets were acquired from 24 patients with TAAD. Manual segmentation and annotation of the CT images was used as the ground-truth. Two-dimensional (2D) nnU-Net and three-dimensional (3D) nnU-Net architectures with Dice- and cross entropy-based loss functions were utilized to segment the true lumen (TL), false lumen (FL), and intimal flap on the images. Four-fold cross validation was performed to evaluate the performance of the two nnU-Net architectures. Six metrics, including accuracy, precision, recall, Intersection of Union, Dice similarity coefficient (DSC), and Hausdorff distance, were calculated to evaluate the performance of the 2D and 3D nnU-Net algorithms in TAAD datasets. Aortic morphological features from both 2D and 3D nnU-Net algorithms were quantified based on the segmented results and compared. Results: Overall, 3D nnU-Net architectures had better performance in TAAD CT datasets, with TL and FL segmentation accuracy up to 99.9%. The DSCs of TLs and FLs based on the 3D nnU-Net were 88.42% and 87.10%. For the aortic TL and FL diameters, the FL area calculated from the segmentation results of the 3D nnU-Net architecture had smaller relative errors (3.89–6.80%), compared to the 2D nnU-Net architecture (relative errors: 4.35–9.48%). Conclusions: The nnU-Net architectures may serve as a basis for automatic segmentation and quantification of TAAD, which could aid in rapid diagnosis, surgical planning, and subsequent biomechanical simulation of the aorta.

## 1. Introduction

Aortic dissection is a severe cardiovascular disease caused by a tear in the intimal layer of the aorta, which results in the separation of the layers of the aortic wall and congestion in the tunica media, leading to blood flow deficiency and even mortality. When the dissection involves the ascending aorta, it is defined as type A aortic dissection (TAAD), which is usually managed with an emergency open-chest surgery [1]. Computed tomography (CT) is a common imaging modality used for screening and evaluating patients with aortic dissection in clinical settings [2,3], as it can provide important morphological information on the dissected aorta to guide the clinical diagnosis and treatment decision-making of acute aortic dissection.

The diameter of the ascending aorta is one of the key morphological features for aortic dissection management. The guidelines of the European Society of Cardiology on the diagnosis and treatment of aortic diseases recommend prophylactic replacement of the ascending aorta to prevent acute TAAD when the diameter of the ascending aorta is larger than 55 mm [4,5]. Furthermore, the diameter of the dissected ascending aorta serves as a basis for selecting artificial aortic grafts of the right size in the aortic replacement operation [6]. Other morphological information, including the true lumen (TL), false lumen (FL), and intimal flap, also have important prognostic significance [7]. The intimal flap fenestration is commonly implemented to depressurize the FL by puncture of the intimal flap from the TL into the FL for patients with malperfusion syndrome [4]. Hence, accurately segmenting these morphological features of TAAD from CT images is vital for assessment of aortic dissection.

A large number of cross-sectional images of the dissected aorta are generated from CT scans. The manual segmentation of these images is too time-consuming to meet the clinical need. Recently, deep learning approaches were used to complete the task [8]. Li et al. applied a 2D cascaded convolutional network to extract the contours of the aorta and TL from contrast-enhanced CT datasets of 45 patients with aortic dissection. Their proposed method showed good performance, with Dice similarity coefficients (DSCs) of 0.989 for the aorta and 0.925 for the true lumen [9]. This was the first study to target aortic dissection segmentation using deep learning. However, few studies on the segmentation of TAAD exist in the current literature, compared to type B aortic dissection (TBAD, dissection that does not involve the ascending aorta). Many excellent deep learning methods were proposed to segment TBAD [10,11,12,13]. Hahn et al. proposed a stepwise convolutional neural network (CNN)-based approach to segment the TL and FL using 153 CT datasets from 45 patients with TBAD, with the DSC ranging from 0.87 to 0.90 for segmenting the TL and FL [11]. Considering the significant prognostic value of false lumen thrombosis for predicting late adverse events, Wobben et al. utilized 3D residual U-Net-based models for segmenting the TL, FL, and false lumen thrombosis [12]. Their best results for TL and FL segmentation achieved median (interquartiles) DSCs of 0.85 (0.77 0.88) and 0.84 (0.82 0.87), respectively. Xiang et al. proposed a flap-attention-based deep learning approach for segmenting the TL and FL (flap simplified to the boundary between the TL and FL) of TBAD, with DSCs of 91.1% and 88.4% [14]. In addition to the segmentation of the TL or TL + FL, some studies focused on the segmentation of the intimal flap [15,16]. Lyu et al. constructed a CNN-based two-step algorithm to capture the aortic structure and intimal flaps without distinguishing the TL and FL, and they achieved an average DSC of over 92% [16]. U-Net, as the deep learning architecture of a pixel-level classification, is well suited for segmentation tasks and is widely used in image segmentation [17]. Cheng et al. introduced a semantic segmentation algorithm based on the U-Net framework to segment the aortic TL using contrast-enhanced CT images obtained from 20 patients with TAAD, with 85.00% accuracy (90.00% sensitivity, 80.00% specificity) [18].

nnU-Net (no new U-Net) is a state-of-the-art network architecture for biomedical image segmentation and is extensively applied to the segmentation of many different organs and tissues, including the lung and heart; it can automatically adapt its pre-processing strategies and network architecture to a given image dataset without additional manual tuning [19]. However, similar designs based on nnU-Net have not been employed to segment TAAD on CT images. For TAAD, segmentation and morphological quantification of the dissected ascending aorta, including the TL, FL, and intimal flap, are essential for surgical interventions such as aortic valve replacement or ascending aortic replacement [20]. In this study, nnU-Net was adopted to segment contrast-enhanced CT data from 24 patients with TAAD to characterize the TL, FL, and intimal flap, and quantify the aortic morphology to examine its performance. Aortic morphological factors, including the diameter of the dissected aorta, the thickness of the intimal flaps, and the areas of the TL and FL, were measured. These are crucial indicators for surgical planning and follow-up studies, such as computational simulation and modeling.

## 2. Materials and Methods

### 2.1. Data Acquisition and Processing

**Figure 1:**
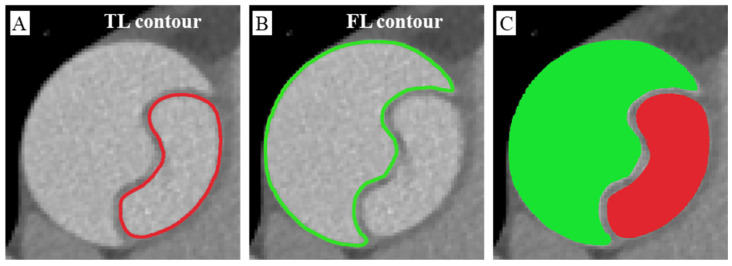
Manual annotation procedure. (**A**) The manual TL contour; (**B**) the manual FL contour; (**C**) the labeled areas of TL and FL in aorta. Red color, TL; green color, FL.

A total of 24 patients with TAAD (20 males and 4 females, mean age ± standard deviation: 56.3 ± 9.4) who underwent surgical repair of the aorta following Sun’s procedure were enrolled at Jiangsu Province Hospital, with informed consent obtained [20]. The diagnosis of aortic dissection was evaluated by pre-operative CT examinations using a CT system with 64-row detectors (SOMATOM Definition AS, Siemens AG). All patients were scanned through CT after injection of intravenous contrast and with a 1.25 mm slice width. One CT sequence was selected from each patient to perform subsequent image segmentation. The CT data were collected following the protocol approved by the Medical Ethics Committee of Jiangsu Province Hospital (approval number: 2022-SR-730). The original images in DICOM format with a slice size of 512 × 512 pixels were converted to the NIfTI format as the standard data format for the segmentation task. Manual annotation was performed to label the TL and FL regions of the aorta on the CT slices (see Figure 1). The unlabeled region on the CT slice was perceived as the background. Of note, the intimal flap region was not labeled at this stage, and would be extracted after image segmentation using deep learning. For the consistency and reliability of manual annotation, the labeling was accomplished by three experts following the standard procedure described in Isensee et al. and Yao et al. [13,19]. The 24 CT datasets were randomly divided into two groups. The segmentation task of two groups was assigned to two different experts, and then checked by each other, slice-by-slice, after segmentation and annotation, with the disagreement and inconsistency between the two experts resolved by the third expert. Manual annotation took approximately 2.5 h per dataset. The conversion of medical image format and manual labeling were executed with the open source ITK-SNAP software (version 4.0, http://www.itksnap.org/ (accessed on 1 June 2023)) [21].

### 2.2. Segmentation Network Architecture

**Figure 2:**
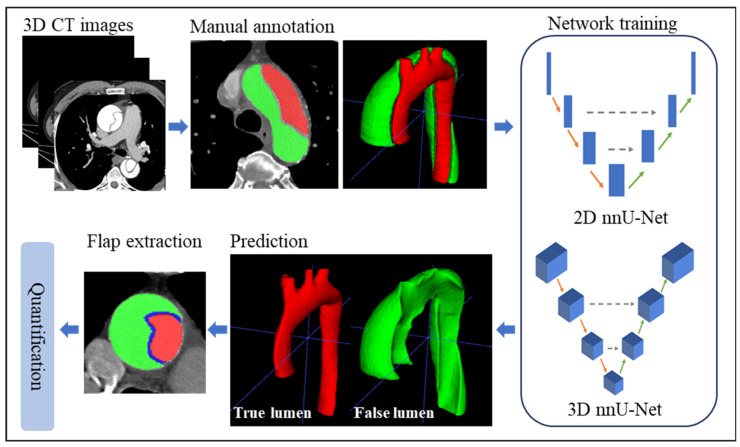
Basic flowchart of TAAD segmentation based on nnU-Net architectures.

Both two-dimensional (2D) and three-dimensional (3D) nnU-Net frameworks with Dice and cross entropy-based loss functions were utilized to segment the aortic region of contrast-enhanced CT images from 24 patients with TAAD, and the performances of these two networks were compared. The nnU-Net architecture integrates with pre- and post-processing. For pre-processing, data augmentation contains rotations, scaling, Gaussian noise and blur, brightness, contrast and simulation of low resolution, gamma correction, and mirroring. Intensity normalization was performed on all the CT images by clipping them to an intensity range between 0.5 and 99.5 percentiles, and then the images were normalized using a global foreground mean and a standard deviation. All images were resampled to 1.25 × 0.8242 × 0.8242 mm. The stochastic gradient method with Nesterov’s momentum was used to optimize the learning rate, in which the initial values of the learning rate and Nesterov’s momentum were set at 0.01 and 0.99, respectively. Then, the learning rate was decayed throughout the training process, following the ‘poly’ learning rate policy: (1 − epoch/epoch_max_)^0.9^. More details can be found in [19]. The number of epochs was set at 600 according to the performance of the model on the validation set. All experiments were conducted with Python 3.8.0, Pytorch 2.2.1 and NVIDIA CUDA 11.8 on NVIDIA RTX 4090 GPU with 24 GB of video memory. The batch size, patch size, and epoch time were 12, 512 × 512, and 34 s (per epoch) for the 2D nnU-Net network, and were 2, 56 × 224 × 192, and 42 s (per epoch) for the 3D nnU-Net network, respectively. The training times per fold of the 2D and 3D nnU-Net networks were 5.8 h and 7.3 h. The basic flowchart of TAAD segmentation using the 2D and 3D nnU-Net is shown in Figure 2.

### 2.3. Validation and Performance Evaluation

Twenty-four CT datasets were randomly divided into four groups. Four-fold cross validation was employed, with any three out of four groups used as training data, while the remaining one group was utilized as the testing data. Six metrics, including accuracy, precision, recall, Intersection of Union (IoU), Dice similarity coefficient (DSC), and Hausdorff distance (HD), were calculated to evaluate the performance of 2D and 3D nnU-Net algorithms on the TAAD dataset. The six metrics were calculated according to the following formulas:

accuracy = (TP + TN)/(TP + TN + FP + FN),(1)

precision = TP/(TP + FP),(2)

recall = TP/(TP + FN),(3)

IoU = TP/(TP + FP + FN),(4)

DSC = 2 × TP/(2 × TP + FP + FN),(5)

HD = max (h(GT, UN), h(UN, GT)),(6)

where TP is the number of true-positive outcomes, FP is the number of false-positive outcomes, TN is the number of true-negative outcomes, FN is the number of false-negative outcomes, GT is ground-truth, UN is the nnU-Net method result, and h(GT, UN) = max_g∈GT_ min_u∈UN_‖g − u‖, ‖·‖ is the L2 norm. The six metrics were calculated using the TP, TN, FP, FN, GT, and UN of the CT images on a per-voxel basis.

### 2.4. Post-Processing and Quantification of Aortic Morphological Information

Each CT slice was segmented by both 2D and 3D nnU-Net architectures to output the segmented TL and FL regions. Then, the contours of the TL and FL were extracted in segmented CT images with TL and FL output labels, respectively, using the active contour models [22]. To obtain the contours of the whole aorta, active contour models were applied again to the segmented image by treating TL + FL as the same labeling region. The intimal flap region was determined by the remaining region after removing the TL and FL in the whole aortic area. After that, the cross-sectional whole aorta was categorized into three regions, including TL, FL, and intimal flap. The maximum diameter of the aorta, the areas of the TL and FL, and the average thickness of the flap were quantified for clinical assessment. Relative errors from both nnU-Net architectures were calculated and compared to determine which one had better performance in quantifying aortic morphological information.

### 2.5. Statistical Analysis

All output segmentation results of the CT slices were gathered to present patient-level results. The Shapiro–Wilk test was used to test the normality of the patient-level results. The differences of six metrics between 2D and 3D nnU-Net networks all obey normal distribution. Therefore, the differences between the results of the 2D and 3D nnU-Net networks were compared using the paired *t*-test. A *p*-value of <0.05 is considered as statistically significant. Statistical analysis was performed with MATLAB (MathWorks Inc., Natick, MA, USA).

## 3. Results

### 3.1. Performance of nnU-Net Architectures

**Figure 3:**
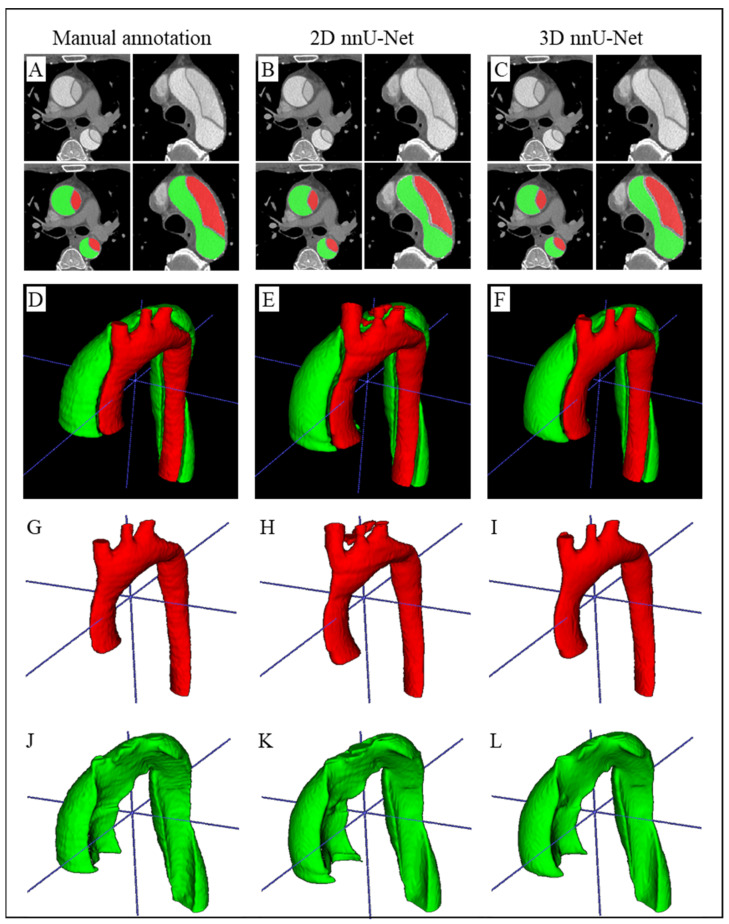
The 3D visualization of ascending aorta from manual annotation, 2D nnU-Net, and 3D nnU-Net. The four subgraphs in the first row (**A**–**C**) are two CT images of ascending aorta and arch and corresponding segmentation. (**D**–**F**) are the 3D visualization of ascending aorta from manual annotation, and prediction of 2D and 3D nnU-Net. (**G**–**I**) are the 3D visualization of TL. (**J**–**L**) are the 3D visualization of FL. Red region, TL; green region, FL.

The TL and FL regions of the 2D and 3D nnU-Net architectures were used to calculate the six metrics at the patient level: accuracy, precision, recall, IoU, DSC, and HD. The statistics of the six metrics at the patient level were listed in Table 1. For TL prediction, the 3D nnU-Net was significantly higher in accuracy, precision, IoU, DSC, and HD, compared to the 2D nnU-Net (all *p*-values < 0.05). For FL prediction, the 3D nnU-Net was significantly higher only in accuracy, with *p*-value 0.0380. All the prediction slices from the 24 CT datasets were stacked into a 3D vessel structure for aortic geometry visualization (see Figure 3D–L). The prediction results of the 3D nnU-Net architecture had better smoothness. Overall, the 3D nnU-Net architecture had better performance in TAAD CT datasets, and the segmentation of the TLs was better than the segmentation of the FLs for all metrics. Figure 3 shows the output segmentation of the 2D and 3D nnU-Net architectures from one sample patient, including segmentation of two CT slices, and the 3D structures of the TLs and FLs.

### 3.2. Quantification of Aortic Morphological Information

**Figure 4:**
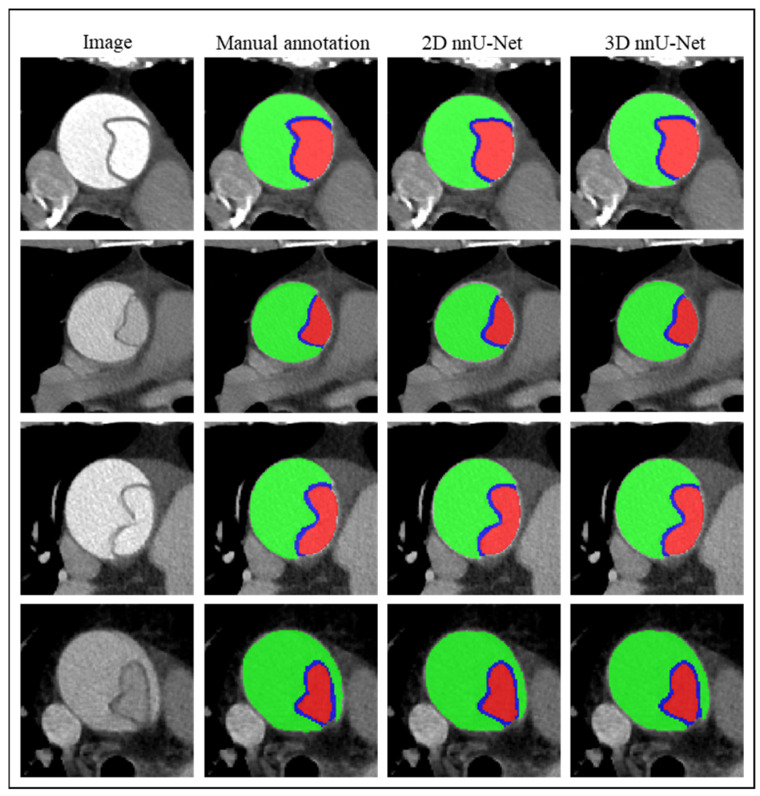
Three regions in the cross-section of ascending aorta segmented by ground-truth, 2D nnU-Net, and 3D nnU-Net from four sample patients. Red region, TL; green region, FL; blue region, intimal flap.

Figure 4 shows the cross-section of the ascending aorta after extracting the intimal flap from the output segmentation. The region between the TL and FL was characterized as the intimal flap. CT slices containing the TL, FL, and intimal flap in the section of the ascending aorta were chosen to calculate the diameter, area, etc. The outputs from twenty-one patients were adopted to quantify the morphological features of dissected aortas, while the other three patients were not processed due to the tiny size of the tear in the ascending aorta region compared to the large dissection size in the aortic arch region. The cross-section images of the aortic arch (Figure 3A) could not provide accurate aortic morphology, such as aortic diameter and the TL and FL diameters of the ascending aorta, and thus the three patients were not included in the analysis of the quantifying aortic morphological features. The average values of the aortic morphological factors were used to quantify the aortic morphology of the ascending aorta for patients with TAAD. Table 2 shows the quantifications of TL, FL, and intimal flap according to manual annotation (ground-truth), and the output segmentation of the 2D nnU-Net and 3D nnU-Net, respectively. Relative error was adopted to compare the quantification of the TL, FL, and intimal flap from the two architectures, with manual annotation as the reference. The TL area and intimal flap thickness calculated from the output segmentation of the 2D nnU-Net architecture had smaller relative errors: 6.42% and 12.55%. The aortic, TL, and FL diameters, and the FL area calculated from the output segmentation of the 3D nnU-Net architecture all had smaller relative errors (3.89–6.80%), compared to the 2D nnU-Net architecture (relative errors: 4.35–9.48%).

## 4. Discussion

### 4.1. Segmentation Based on Artificial Intelligence

Medical image segmentation plays an increasingly important role in disease diagnosis, assessment, and treatment. Contrast-enhanced CT imaging has significant clinical diagnostic value and advantages for TAAD. The large number of CT images not only bring a wealth of aortic information, but also pose a huge burden on image segmentation processing. TAAD is a dangerous and urgent aortic disease, and it is unable to withstand the time-consuming manual segmentation in a clinical setting. With the rapid development of artificial intelligence, deep learning has been applied to a variety of medical fields, such as image segmentation, disease screening, identification, and prediction [8,23]. The nnU-Net proposed by Isensee et al. is a self-configuring method for deep learning-based segmentation, and is the clear winner of the Medical Segmentation Decathlon challenge [19,24]. The CT datasets of TAAD and the task segmentation of the TL and FL are applicable to nnU-Net due to the versatility, ease of use, and robustness of this network. In this study, the 2D and 3D nnU-Nets were employed to segment the aortic TLs and FLs, using contrast-enhanced CT images obtained from 24 patients with TAAD, and they achieved over 99% accuracy. Particularly, precision and recall based on the 3D nnU-Net exceeded 88.57% and 86.94%, respectively. Cheng et al. compared the performance of seven deep learning algorithms in a TL segmentation task of the TAAD dataset. The results showed that the accuracy and DSCs of the seven algorithms ranged from 93.88% to 99.42%, and 80.08% to 90.58%, respectively [18]. The performance of the 3D nnU-Net in the TL and FL segmentation in this study was good, with accuracy up to 99.9%. Additionally, the DSCs of the TLs and FLs based on the 3D nnU-Net in our TAAD dataset are 88.42% and 87.10%. Compared to DSC values ranging from 84% to 90% when segmenting the TLs and FLs based on the CNN algorithm and the 3D residual U-Net using the TBAD dataset [10,12], the performance of the 3D nnU-Net in TL and FL segmentation is still excellent in the TAAD dataset. These TAAD datasets included a variety of tear configurations, as shown by the different false lumen shapes on cross-sectional CT images, which should be a good representation of the patient population of TAAD, but there still needs to be more validation for these results when generalizing to a larger dataset.

### 4.2. Significance of Quantification of Aortic Morphological Information

The quantification of dissected aorta morphology is an integral part of the diagnostic workflow in clinical routines. It provides fundamental information for the selection of clinical treatment options and important aortic structures for subsequent biomechanical simulation. The diameter of the ascending aorta is an important morphological factor in diagnosis and aortic replacement for TAAD. Among aortic, TL, and FL diameters, the 2D and 3D nnU-Net architectures performed best in quantification of the FL diameter, with the average of relative error less than 4.35%, and worst in quantification of the TL diameter, with the average of relative error above 4.89%. The FL area and intimal flap thickness had worse performances from the two nnU-Net architectures. This may be affected by the false lumen thrombus, which is regarded as the false lumen region in manual annotation because of the small number of thrombi in this study. Accurate quantification could contribute to diagnosis and treatment, and it also provides essential information for the computational simulation of aortic biomechanics. Since the pathogenesis of TAAD is still obscure, many studies focused on the biomechanical modeling of the aorta to investigate the biomechanical mechanism of TAAD [25,26]. The TL, FL, and intimal flap are the structures of the dissected aorta. The segmentation of the three regions in the aorta is necessary for more accurate biomechanical simulation. From the aspect of clinical utility, streamlining the whole segmentation process is necessary. Our work has completed the FL/TL segmentation, flap extraction, quantification, and visualization of TAAD. If these discrete efforts are integrated into clinical software, it would be beneficial to the diagnosis of aortic dissection.

### 4.3. Limitations

One major limitation of this study is that the segmentation of the false lumen thrombus was not performed. For simplification, the false lumen thrombus was treated as the false lumen region, and not regarded as an additional region different from the TL, FL, and intimal flap regions. The false lumen thrombus should be treated as a different label from the existing FL and TL labels, and its segmentation should be taken into consideration in follow-up studies. Another limitation is the small sample size of CT datasets studied. Large-scale studies with more CT datasets are needed to validate and improve the significance of the prediction method.

## 5. Conclusions

This study demonstrates that deep learning is a valuable, broadly applicable tool for segmentation and quantification of the aorta with TAAD in a clinical setting. The nnU-Net architecture may serve as a basis for automatic segmentation and quantification of TAAD, which could aid in rapid diagnosis, surgical planning, and subsequent biomechanical simulation of the aorta.

## Figures and Tables

**Table 1:** TL and FL segmentations using 2D and 3D nnU-Net architectures: mean and standard deviation values of six metrics for 24 TAAD patients.

Net	Label	Accuracy (%)	Precision (%)	Recall (%)	IoU (%)	DSC (%)	HD
2D	TL	99.90 ± 0.07	86.28 ± 11.42	84.44 ± 11.94	74.51 ± 13.68	84.68 ± 9.56	24.10 ± 10.92
	FL	99.85 ± 0.09	86.34 ± 9.66	83.79 ± 13.30	73.56 ± 12.25	84.17 ± 8.84	30.40 ± 19.51
3D	TL	99.92 ± 0.06	89.71 ± 11.18	87.94 ± 9.11	80.20 ± 12.71	88.41 ± 8.97	19.40 ± 10.10
	FL	99.89 ± 0.07	88.57 ± 10.03	86.94 ± 12.51	78.19 ± 13.13	87.10 ± 9.39	28.68 ± 20.49
*p*-value	TL	0.0225	0.0483	0.0845	0.0223	0.0266	0.0280
	FL	0.0380	0.1853	0.2107	0.1001	0.1592	0.4594

*p*-value from paired *t*-test.

**Table 2:** TL, FL, and intimal flap quantifications according to manual annotation (ground-truth), output segmentation of 2D nnU-Net, and output segmentation of 3D nnU-Net, respectively: the diameters of whole aorta, TL, and FL; areas of TL and FL; and intimal flap thickness.

		Aortic Diameter (mm)	TL Diameter (mm)	FL Diameter (mm)	TL Area (mm^2^)	FL Area (mm^2^)	Flap Thickness (mm)
GT	Ave.	49.71	36.07	47.32	566.83	927.47	2.35
2D	Ave. RE Ave.	48.91 4.58%	36.02 5.55%	45.60 4.35%	566.73 6.42%	869.15 9.48%	2.46 12.55%
3D	Ave. RE Ave.	49.61 4.42%	36.47 4.89%	46.74 3.89%	577.93 6.94%	892.84 6.80%	2.41 14.08%

Legend: GT, ground-truth; Ave., average; RE, relative error; TL, true lumen; FL, false lumen; Flap, intimal flap.

## Data Availability

The datasets presented in this article are not readily available because the data are part of an ongoing study. Requests to access the datasets should be directed to the corresponding author.
